# A novel 2-phase residual U-net algorithm combined with optimal mass transportation for 3D brain tumor detection and segmentation

**DOI:** 10.1038/s41598-022-10285-x

**Published:** 2022-04-19

**Authors:** Wen-Wei Lin, Jia-Wei Lin, Tsung-Ming Huang, Tiexiang Li, Mei-Heng Yueh, Shing-Tung Yau

**Affiliations:** 1 Department of Applied Mathematics, National Yang Ming Chiao Tung University, Hsinchu, 300 Taiwan; 2 Department of Mathematics, National Taiwan Normal University, Taipei, 116 Taiwan; 3 Nanjing Center for Applied Mathematics, Nanjing, 211135 People’s Republic of China; 4 School of Mathematics and Shing-Tung Yau Center, Southeast University, Nanjing, 210096 People’s Republic of China; 5 Department of Mathematics, Harvard University, Cambridge, USA

**Keywords:** Computational biology and bioinformatics, Machine learning

## Abstract

Utilizing the optimal mass transportation (OMT) technique to convert an irregular 3D brain image into a cube, a required input format for a U-net algorithm, is a brand new idea for medical imaging research. We develop a cubic volume-measure-preserving OMT (V-OMT) model for the implementation of this conversion. The contrast-enhanced histogram equalization grayscale of fluid-attenuated inversion recovery (FLAIR) in a brain image creates the corresponding density function. We then propose an effective two-phase residual U-net algorithm combined with the V-OMT algorithm for training and validation. First, we use the residual U-net and V-OMT algorithms to precisely predict the whole tumor (WT) region. Second, we expand this predicted WT region with dilation and create a smooth function by convolving the step-like function associated with the WT region in the brain image with a $5\times 5\times 5$ blur tensor. Then, a new V-OMT algorithm with mesh refinement is constructed to allow the residual U-net algorithm to effectively train Net1–Net3 models. Finally, we propose ensemble voting postprocessing to validate the final labels of brain images. We randomly chose 1000 and 251 brain samples from the Brain Tumor Segmentation (BraTS) 2021 training dataset, which contains 1251 samples, for training and validation, respectively. The Dice scores of the WT, tumor core (TC) and enhanced tumor (ET) regions for validation computed by Net1–Net3 were 0.93705, 0.90617 and 0.87470, respectively. A significant improvement in brain tumor detection and segmentation with higher accuracy is achieved.

## Introduction

With the rapid development of convolutional neural network (CNN) architecture design, deep CNNs have undoubtedly become one of the most widely used artificial intelligence techniques for object detection, feature extraction, image classification, and segmentation in medicine and computational science. A deep CNN consists of three key parts, namely, input data, computational steps, and a model. The last two items include the design of the optimization algorithm and the training of the model structure weights. The former is primarily driven by feeding large quantities of data into CNNs to develop more powerful prediction systems. Over the past decade, innovations in high-performance computing, such as graphics processing unit (GPU) accelerators, have driven powerful advances in deep learning. However, due to the GPU memory constraint, it is difficult to train models for large-scale 3D image data; therefore, the computation of these models becomes extremely expensive and inefficient. Recently, to overcome the difficulty of large-scale 3D image data input, a random sampling technique with several filters has been proposed to cover the entire region of the 3D image. Although this approach is advantageous in addressing insufficient GPU memory, it requires more voxels to cover the original image and may lose the connectivity information of the 3D global image. Thus, properly addressing the geometric structure is a crucial task for 3D image segmentation.

In recent years, two benchmark datasets, the Medical Segmentation Decathlon 2018 (MSD2018)^1,2^ and Brain Tumor Segmentation (BraTS) 2020^3–5^ datasets, which contain 484 and 369 labeled 3D brain image samples for brain tumor segmentation, respectively, have provided a challenging platform and have attracted enormous attention and interest from researchers in this field. The brain samples were scanned with four modalities, namely, fluid-attenuated inversion recovery (FLAIR), T1-weighted (T1), T1-weighted contrast-enhanced (T1CE), and T2-weighted (T2), by multiparametric magnetic resonance imaging (mpMRI). The challenge is the evaluation of state-of-the-art methods for the task of brain tumor segmentation of whole tumor (WT, labeled {2,1,4}), tumor core (TC, labeled {1,4}), and enhanced tumor (ET, labeled {4}) regions in the human brain. To address this issue, in the early years, random forest algorithms and machine learning techniques were used to perform image classification^6–8^ and segmentation^6,9–11^. In 2021, Biratu et al. provided a significantly comprehensive survey^12^ of three model techniques, including region growing^13^, shallow machine learning^14^, and deep learning^15^, for brain tumor segmentation and classification. Later, BraTS 2021^3,4,16^ was expanded to include a large number of new brain samples in the database, providing 1251 labeled samples for training and 219 unlabeled samples for validation. Subsequently, CNN structures with two layers^17^ and eight layers^18^ were proposed and made good progress in brain tumor segmentation. Then, a more sophisticated multiple CNN architecture, called the U-net model, was first developed in^19^ and improved in^20^ by assembling two full CNNs and U-net. The merits of applying the U-net model to the challenge of MSD 2018^1,2^ were first proposed by Isensee et al.^21^. In 2018, a variant U-net, called the residual U-net^22^ (ResUnet), was proposed to enhance the segmentation accuracy. By combining U-net and residual units^23,24^, ResUnet simplifies the training of deep networks, promotes the dissemination of information via a large number of skip connections, and implements a network designed with leaner parameters and superior performance. Therefore, we adopt ResUnet as the U-net architecture used for the model training and prediction in this paper.

For the study of brain tumor segmentations, preprocessing to effectively represent large quantities of input data for CNNs is crucial. For example, taking an irregular 3D physical brain image obtained from an MRI, which is generally composed of 1.5 million voxels, by randomly selecting several cubes (e.g., 16 cube filters were used by Isensee et al.^25^) with seamless coverage to overlay the irregular brain image is a natural way to fit the input format of tensors for a U-net architecture. Nevertheless, the random sampling technique may lose the global information of the brain image, and it increases the quantity of input data. On the other hand, an efficient two-stage optimal mass transportation (2SOMT) algorithm, newly proposed by Lin et al.^26^, was designed to first transform an irregular 3D brain image into a unit ball and then into a cube with minimal distortion and transport costs. This strategy can greatly reduce the capacity of input data and retain the global information of the 3D brain image, so the existing computing resources can be effectively used to attain the expected result. However, the 2SOMT did not fully use the merits of the density distribution of the brain image so that a U-net algorithm could not predict the tumor region more accurately. In addition, 2SOMT may produce more conversion loss between transformations from a brain to a ball and then to a cube. Thus, we are motivated to consider directly transforming an irregular brain image into a cube with a more precise density function to detect possible tumor regions so that a U-net algorithm is better positioned to learn to label the segmentation.

Optimal mass transportation (OMT) is a very old optimization problem that was raised by Monge in 1781 (see^27^ for details) to find an optimal solution that minimizes the transport cost and preserves the local mass ratios between two spaces. The existence and uniqueness of a solution to the OMT problem was proven by Kantorovich^27^ by relaxing the probability measure with a joint probability distribution. The regularity condition for the solution of the OMT problem was first shown by Caffarelli^11^, and an elegant theoretical survey paper “Optimal Transport: Old and New”, which summarized the achievements of predecessors, was published by Villani^28^. For numerical methods, Brenier^10^ proposed an alternative scheme for solving the OMT problem with a quadratic cost function for a special class of convex domains. Based on Brenier’s approach and the variational principle^29^, Su et al.^30^ developed a volume-preserving parameterization from a 3-manifold $\mathcal {M}$ with a spherical boundary to a unit ball $\mathcal {B}^3$. Recently, Yueh et al.^31^ proposed a novel algorithm to compute a volume-preserving parameterization from $\mathcal {M}$ to $\mathcal {B}^3$ by modifying the denominators of the coefficients of the corresponding Laplacian matrix by imposing the local volume stretch factor at each iteration step and adopted the projected gradient method (PGM) combined with the homotopy technique in^32^ to find the OMT map between $\mathcal {M}$ and $\mathcal {B}^3$. In addition, the 2SOMT procedure from $\mathcal {M}$ to $\mathcal {B}^3$ and from $\mathcal {B}^3$ to a cube was developed by Lin et al.^26^ and applied prior to ResUnet training and inference in 3D brain tumor segmentation.

In this paper, we study the applicability of mapping an irregular 3D image (i.e., a human brain) to a canonical domain (i.e., a cube or a cuboid), which minimizes the transport cost and preserves the local mass ratios. First, based on the homotopy technique, a direct one-stage OMT approach from a 3-manifold $\mathcal {M}$ with a genus-zero boundary to a cube is developed for 3D ResUnet training and inference to improve the higher conversion loss of 2SOMT^26^ from $\mathcal {M}$ to $\mathcal {B}^3$ and $\mathcal {B}^3$ to a cube. Thus, we can construct a one-to-one correspondence between the input data of irregular images and the associated cubic tensors. With slight conversion loss between OMT maps, the usage of the capacity of the training data of the 3D ResUnet model is greatly reduced, and it is our belief that 3D ResUnet training can easily find a local minimum and achieve better performance.

Next, we propose a two-phase ResUnet with OMT (2P-ResUnet-OMT) algorithm utilizing the density distribution of brain tumor features and train four related networks to detect tumor regions and segment tumor labels. Given an irregular 3D brain, in Phase I, we first construct the associated density map at each vertex according to the normalized contrast-enhanced histogram equalization (CEHE) grayscale values of the FLAIR modality of a brain image by MRI. Then, we compute OMT maps from brain images to cubes for the training set and train Net0 by the ResUnet algorithm for the detection of possible tumor regions. In fact, there are no clues at the beginning; the CEHE grayscales of FLAIR, which typically reflect the distribution of WT, should be an effective way to detect tumor regions. Next, we covered these possible tumor regions with 5 voxels with morphological dilation. In Phase II, because $\text{ ET }\subset \text{ TC }\subset \text{ WT }$, we construct a smooth density function by convolving the step-like function with $\exp (\text {FLAIR})$ on the expanded WT region and 1.0 on the others, with a $5\times 5\times 5$ blur box tensor. We remesh the tetrahedron with finer meshes in the higher density region in the brain so that the target tumor region can be enlarged in the cube by OMT and better viewed and learned by ResUnet. We then train Net1 for WT, Net2 for TC and Net3 for ET by ResUnet. In practice, for the testing issue, Net0 can help by first detecting a WT region as much as possible. As discussed above, by covering this region with 5 voxels using dilation and creating a similar smooth density function with finer meshes on the raw brain image, we compute the corresponding OMT map and call Net1–Net3 combined with ensemble voting postprocessing to make the final label prediction and image segmentation.

### Contribution

The 2P-ResUnet-OMT procedure transforms an irregular 3D brain image into a cube with density estimates and mesh refinement to fit the input format of the ResUnet algorithm. Unlike the previous methods, 2P-ResUnet-OMT minimizes the transport cost and preserves the global features of input data to surpass the other methods. The main contributions of this paper are summarized as follows.

1. Our proposed 2P-ResUnet-OMT algorithm transforms an irregular 3D brain image into a cube to satisfy the input format of ResUnet while preserving the local mass ratios between two domains and minimizing the transport cost and the distortion. These advantages for 2SOMT were highlighted in^26^. However, 2SOMT did not make full use of estimating the distribution of the density function, so ResUnet could not infer the target object accurately. 2P-ResUnet-OMT fully grasps the distribution of the associated density function to create an effective OMT map from an irregular 3D domain to a cube and provides it to ResUnet for training a high-performance prediction network. 2P-ResUnet-OMT inherits the advantages of 2SOMT in that it needs to use only a cube to represent an irregular 3D brain image without losing the most important global features and conversion accuracy. In this way, the computational cost and the computer environment can be greatly economized during ResUnet training and used for data augmentation, which exactly considers the limitation of the memory capacity.
2. One of the characteristics of the OMT map is to preserve the local mass ratios. With this peculiar feature, in Phase II of 2P-ResUnet-OMT, we apply mesh refinement on the expanded WT region detected during Phase I. The mesh refinement technique can increase the number of tetrahedrons in a specific region in the brain and enlarge the portion of volume appearing in the target domain; that is, using the ResUnet algorithm is similar to using a magnifying glass to view and learn how to mark the segmentation labels well. The numerical experiment with the trained Net1–Net3 models combined with ensemble voting shows that the Dice scores of validation for WT, TC and ET can reach 0.93705, 0.90617 and 0.87470, respectively; hence, this approach significantly boosts the accuracy of brain tumor detection and segmentation.
3. The OMT approach must convert the labels predicted by ResUnet to a brain image; therefore, to evaluate the Dice score more precisely, we propose a new conversion technique with ensemble voting postprocessing to convert the predicted labels on the cube back to each voxel of the brain by using the multiple values on the cube validated by various models to precisely evaluate the labels corresponding to voxels in the brain image. The expressively high validation Dice scores on the BraTS 2021 validation data suggest that using a cube to represent an irregular 3D brain image by OMT is indeed an innovative idea and the most streamlined approach for CNN training and prediction.

This paper is organized as follows. In “Discrete OMT problems and cubic OMT maps”, we introduce the discrete OMT problem and the spherical-cubic area-measure-preserving and cubic volume-measure-preserving OMT maps. In “Two-phase ResUnet with OMT for training and validation”, we propose a two-phase ResUnet model with OMT maps for training and validation. For the evaluation of high Dice scores, we develop an effective conversion technique to convert the predicted labels on the cube back to the brain image using all related probability information corresponding to each voxel in the brain image. In “Results and discussions”, we show the improvement in the Dice score obtained by the ResUnet models in Phase II with mesh refinements on the expanded WT region provided by Phase I and the ensemble voting postprocessing for the label evaluation. Finally, concluding remarks are given in “Conclusions”.

## Discrete OMT problems and cubic OMT maps

Let $\mathcal {M}$ be a simplicial 3-complex that describes an irregular 3D brain image with a genus-zero boundary. $\mathcal {M}$ is generally composed of sets of vertices $\mathbb {V}(\mathcal {M})$, edges $\mathbb {E}(\mathcal {M})$, faces $\mathbb {F}(\mathcal {M})$ and tetrahedrons $\mathbb {T}(\mathcal {M})$. A discrete OMT problem consists of finding a bijective function that maps $\mathcal {M}$ to a canonical simple domain with minimal distortion. The canonical shape could be a ball $\mathcal {B}^3$ or a unit cube $\mathcal {C}^3$. A tensor form is necessary for the input of the U-net algorithm; therefore, a cube or a cuboid is the target domain for $\mathcal {M}$. In this section, we propose a one-stage OMT approach to map $\mathcal {M}$ to $\mathcal {C}^3$.

### Discrete OMT problem

Let $\rho$ be a density map on $\mathbb {V}(\mathcal {M})$. The piecewise linear density functions of $\rho$ on $\mathbb {F}(\partial \mathcal {M})$ and $\mathbb {T}(\mathcal {M})$ are defined by

1 $$\begin{aligned} \rho (\alpha ) = \frac{1}{3} \sum _{i=1}^3 \rho (\hat{v}_i), \quad \rho (\tau ) = \frac{1}{4} \sum _{i=1}^4 \rho (v_i), \end{aligned}$$

respectively, where $\hat{v}_i\in \mathbb {V}(\alpha )$, $\alpha \in \mathbb {F}(\partial \mathcal {M})$, $v_i\in \mathbb {V}(\tau )$, and $\tau \in \mathbb {T}(\mathcal {M})$. Furthermore, we define the local area/volume measures (i.e., local mass) by

2 $$\begin{aligned} a_{\rho }(\hat{v}) := \frac{1}{3}\rho (\hat{v})\sum _{\hat{v}\subset \alpha } |\alpha |, \quad m_{\rho }(v) := \frac{1}{4} \rho (v) \sum _{v\subset \tau } |\tau |, \end{aligned}$$

respectively, where $| \alpha |$ and $| \tau |$ are the area and volume of $\alpha$ and $\tau$, respectively.

Denote

3a $$\begin{aligned} \mathbf {G}_{\rho } = \left\{ g : \partial \mathcal {M}\rightarrow \partial \mathcal {C}^3\,\right| \, \left. \rho (\alpha ) | \alpha | = | g(\alpha ) |, \ \forall \alpha \in \mathbbm{F}(\partial \mathcal {M}) \right\} \end{aligned}$$

and

3b $$\begin{aligned} \mathbf {F}_{\rho } = \left\{ f : \mathcal {M}\rightarrow \mathcal {C}^3\,\right| \, \left. \rho (\tau ) | \tau | = | f(\tau ) |, \ \forall \tau \in \mathbbm{T}(\mathcal {M}) \right\} \end{aligned}$$

as the sets of all area-/volume-measure-preserving (i.e., mass-preserving) piecewise linear maps from $\partial \mathcal {M}$ to $\partial \mathcal {C}^3$ and from $\mathcal {M}$ to $\mathcal {C}^3$, respectively, in which the bijective maps between $\alpha$ and $g(\alpha )$, as well as $\tau$ and $f(\tau )$, are determined by the barycentric coordinates on $\alpha$ and $\tau$, respectively. For given $g \in \mathbf {G}_\rho$ and $f \in \mathbf {F}_{\rho }$, we define the transport costs of *g* and *f*, respectively, by

4 $$\begin{aligned} d_\rho (g) = \sum _{\hat{v} \in \mathbbm{V}(\partial \mathcal {M})} \Vert \hat{v} - g(\hat{v}) \Vert _2^2 a_{\rho }(\hat{v}), \quad c_\rho (f) = \sum _{v \in \mathbbm{V}(\mathcal {M})} \Vert v - f(v) \Vert _2^2 m_{\rho }(v), \end{aligned}$$

where $$a_{\rho }(\hat{v})$$ and $$m_{\rho }(v)$$ are the local area/volume measures at $$\hat{v} \in \mathbb {V}(\partial \mathcal {M})$$ and $$v \in \mathbb {V}(\mathcal {M})$$, respectively, as in (2). The discrete OMT problems on $$\partial \mathcal {M}$$ and $$\mathcal {M}$$ with respect to $$\Vert \cdot 
\Vert _2$$ consist of finding a $$g_\rho ^{*} \in \mathbf {G}_{\rho }$$ and $$f_\rho ^{*} \in \mathbf {F}_{\rho }$$ that solve optimal problems

5 $$\begin{aligned} g_\rho ^{*} = \mathop {{\text {argmin}}}\limits _{g \in \mathbf {G}_{\rho }} d_\rho (g), \quad f_\rho ^{*} = \mathop {{\text {argmin}}}\limits _{f \in \mathbf {F}_{\rho }} c_\rho (f), \end{aligned}$$

where $d_\rho (g)$ and $c_\rho (f)$ are given in (4). Without loss of generality, hereafter, each simplicial 3-complex $\mathcal {M}$ is centralized and normalized so that the center of mass is located at the origin and the mass is one.

### Cubic area-measure-preserving OMT maps

Let $\mathcal {M}$ be a simplicial 3-complex with a genus-zero boundary and of mass one with density functions $\rho :\mathbb {T}(\mathcal {M})\rightarrow \mathbb {R}$ and $\rho :\mathbb {F}(\partial \mathcal {M})\rightarrow \mathbb {R}$ defined on the tetrahedrons of $\mathcal {M}$ and triangles of $\partial \mathcal {M}$, respectively. We define the area-weighted stretch energy on $\partial \mathcal {M}$ with $m=\#\mathbb {V}(\partial \mathcal {M})$. For $\widehat{v}_i\in \mathbb {V}(\partial \mathcal {M})$, $g(\widehat{v}_i) = (g_i^1, g_i^2, g_i^3)$, $i=1,\ldots ,m$, $\mathbf {g}^t=(g_1^t, \ldots , g_m^t)^\top$, $t=1,2,3$ and $\mathbf {g}=[\mathbf {g}^1, \mathbf {g}^2, \mathbf {g}^3]\in \mathbb {R}^{m\times 3}$. The piecewise linear function *g* on $\partial \mathcal {M}$ is given by the barycentric coordinates, *g* is called the induced function by $\mathbf {g}$ and $\mathbf {g}$ is the inducing matrix for *g*. The area-weighted stretch energy^33^ on $\partial \mathcal {M}$ is defined as

6a $$\begin{aligned} E_S(g) = \frac{1}{2} \sum _{t=1}^3 (\mathbf {g}^t)^\top L_S(g) \mathbf {g}^t, \end{aligned}$$

where $L_S(g)$ is the area-weighted Laplacian matrix with

6b $$\begin{aligned}{}[L_S(g)]_{i,j} = {\left\{ \begin{array}{ll} w_{i,j}(g), &{} [v_i,v_j]\in \mathbb {E}(\partial \mathcal {M}),\\ w_{i,i}(g) = -\sum _{\ell \ne i} w_{i,\ell }(g), &{}i=j,\\ 0, &{}\text{ otherwise }, \end{array}\right. } \end{aligned}$$

and

6c $$\begin{aligned} w_{i,j}(g) = -\frac{1}{2} 
\left( \frac{\cot \theta _{i,j}(g)}{\sigma _{g^{-1}}([v_i,v_j,v_\ell ])} + \frac{\cot \theta _{j,i}(g)}{\sigma _{g^{-1}}([v_j,v_i,v_m])}\right) ,\ i \ne j, \end{aligned}$$

where $\theta _{i,j}(g)$ and $\theta _{j,i}(g)$ are two angles opposite to edge $g([v_i,v_j])$ and $\sigma _{g^{-1}}(\alpha ) = \rho (\alpha )|\alpha | / |g(\alpha )|$, for $\alpha \in \mathbb {F}(\partial \mathcal {M})$, is the local area-measure stretch factor.

**Figure 1:**
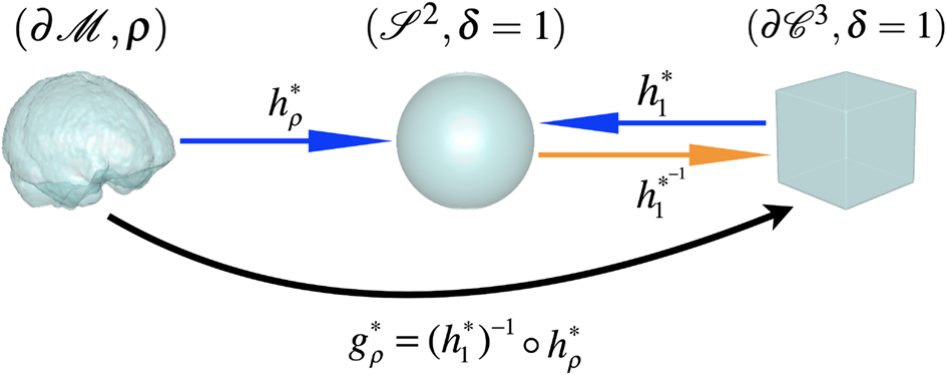
A diagram illustrating the construction of the A-OMT map $g^*_{\rho }$ between $(\partial \mathcal {M}, \rho )$ and $(\partial \mathcal {C}^3, \delta \equiv 1)$.

To compute the cubic area-measure-preserving OMT (A-OMT) map from $\partial \mathcal {M}$ to $\partial \mathcal {C}^3$, we utilize the PGM proposed in^32^, which can be used to efficiently compute the A-OMT maps $h_\rho ^*:\partial \mathcal {M}\rightarrow \mathcal {S}^2$, where $\mathcal {S}^2$ denotes the unit sphere in $\mathbb {R}^3$ and $h_1^*:\partial \mathcal {C}^3\rightarrow \mathcal {S}^2$ ($\rho =1$), respectively. Then, the composition map $g_{\rho }^*=(h_1^*)^{-1}\circ h_\rho ^*:\partial \mathcal {M}\rightarrow \partial \mathcal {C}^3$, as shown in Fig. 1, is the desired A-OMT map. The computational procedure is summarized in Algorithm 1. 
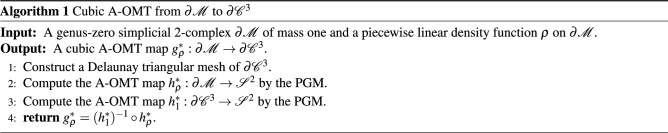

### Cubic volume-measure-preserving OMT maps

In this section, we will develop the OMT algorithm for directly solving the cubic OMT map $f_\rho ^*$, as in (5), from $\mathcal {M}$ to $\mathcal {C}^3$. Let $g_\rho ^*$ be the cubic A-OMT map from $\partial \mathcal {M}$ to $\partial \mathcal {C}^3$ computed by Algorithm 1. We now construct a homotopy $g_\zeta :\partial \mathcal {M}\rightarrow \mathbb {R}^3$ for the boundary maps by

7 $$\begin{aligned} g_\zeta (\widehat{v}) = (1-\zeta ) \widehat{v} + \zeta g_\rho ^*(\widehat{v}),\quad \widehat{v}\in \mathbb {V}(\partial \mathcal {M}), ~ \zeta \in [0,1]. \end{aligned}$$

Then, [0, 1] is uniformly partitioned by $\{0=\zeta _0<\zeta _1<\cdots <\zeta _p=1\}$. Let $L_V(f)$ be the mass-weighted Laplacian matrix with the (*i*, *j*)th coefficient

8 $$\begin{aligned} w_{i,j}(f) = -\frac{1}{9} \sum _{\begin{array}{c} \tau \in \mathbb {T}(\mathcal {M}) \\ {[}{v}_i, {v}_j] \cup [{v}_\ell , v_m] \subset \tau \\ {[}v_i, v_j]\cap [v_\ell ,v_m] = \varnothing \end{array}} \frac{|f([v_i,v_\ell ,v_m])| |f([v_j,v_m,v_\ell ])| \cos \theta _{i,j}^{\ell ,m}(f)}{\rho (\tau )\left| \tau \right| }, \end{aligned}$$

as in^31^, where $\theta _{i,j}^{\ell ,m}(f)$ is the dihedral angle between $f([v_i,v_\ell ,v_m])$ and $f([v_j,v_m,v_\ell ])$ in tetrahedron $f([v_i,v_j,v_\ell ,v_m])$. For $k=1, \ldots , p$, we compute the interior map by solving the linear system

9 $$\begin{aligned} {[}L_V(f^{(k-1)})]_{{\texttt {I}},{\texttt {I}}} \mathbf {f}_{{\texttt {I}}}^{(k)} = -{[}L_V(f^{(k-1)})]_{{\texttt {I}},{\texttt {B}}} {[}g_{\zeta _k}(\widehat{v}){]}_{\widehat{v}\in \mathbb {V}(\partial \mathcal {M})}, \end{aligned}$$

where $f^{(0)}=\mathrm {id.}$, $n = \#(\mathbb {V}(\mathcal {M}))$, ${\texttt {B}}= \{i \,|\, \widehat{v}_i\in \partial \mathcal {M}\}$, and ${\texttt {I}}= \{1, \ldots , n\} \backslash {\texttt {B}}$. The map $f^{(p)}:\mathcal {M}\rightarrow \mathcal {C}^3$ is the desired cubic volume-measure-preserving (V-OMT) map $f_\rho ^*$. The corresponding computational procedure is stated in Algorithm 2. 
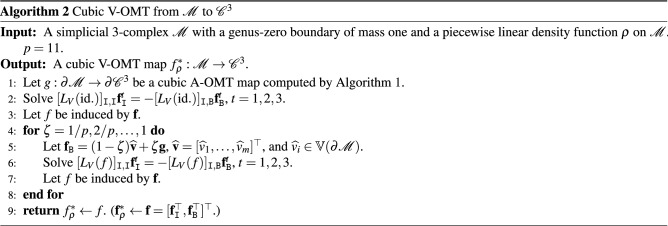


To study the partition number *p* of homotopy in step (9), we define the total mass distortion and the local mass ratio as

10 $$\begin{aligned}d_\mathcal {M}(f) = \sum \limits _{v\in \mathbb {V}(\mathcal {M})}\sum \limits _{\tau \in \mathcal {N}(v)} \left| \frac{\rho (\tau )|\tau |-|f(\tau )|}{4}\right| ,\quad r_f(v) = \sum \limits _{\tau \in \mathcal {N}(v)}\frac{\rho (\tau )|\tau |}{|f(\tau )|}, \end{aligned}$$

respectively, where $\mathcal {N}(v)$ is the set of 1-ring neighboring tetrahedrons of *v*.

## Two-phase ResUnet with OMT for training and validation

A brain image scanned by mpMRI typically provides four modalities, namely, FLAIR, T1, T1CE and T2, with various grayscale values ranging from 0 to 65535 on each voxel of four $240\times 240\times 155$ cuboids, denoted by $\{I_s\}_{s=1}^4$. For a training brain image, let $\mathcal {L}$ denote the $240\times 240\times 155$ labeled cuboid, where $\text {WT}=\{2,1,4\}$, $\text {TC}=\{1,4\}$, $\text {ET}=\{4\}$ and $\{0\}$ for others. The grayscale values on $I_s$ can be normalized by utilizing the Z-score. In practice, these grayscale values are adjusted by CEHE, denoted by $\overline{I}_s$.

A physical brain image $\mathcal {M}$ is contained in $I_s$ and accounts for approximately $12$–$20\%$ of the voxels. Suppose $\mathcal {M}$ is a simplicial 3-complex with a genus-zero boundary composed of tetrahedral meshes representing a brain image. Furthermore, $\overline{I}_1$ records the adapted CEHE grayscales of FLAIR, and in general, the FLAIR modality typically reflects the distribution of $\text {WT}=\{2,1,4\}$; therefore, the adapted CEHE grayscales on the voxel $\overline{I}_1(i,j,k)$ can help with defining the density map on $\mathbb {V}(\mathcal {M})$ by

11 $$\begin{aligned} \rho _\gamma (v) = \exp (\gamma \overline{I}_1(i,j,k)),\quad v\in \overline{I}_1(i,j,k), \end{aligned}$$

where $\gamma$ is a value chosen from the interval [1, 2].

### Two-phase ResUnet with OMT for training

For the given samples in the training set of 3D brain images, we propose a 2P-ResUnet-OMT algorithm with density function estimates to construct an effective input tensor for the ResUnet algorithm. In general, a real brain image contains approximately 1.5 million vertices. Therefore, it is reasonable to cover a brain image with $128^3$ voxels.

The tumor regions of the brain are initially unknown; therefore, in the first phase, we utilize the grayscale of FLAIR to construct the density function in (11) for OMT and train a Net0 by the ResUnet algorithm to detect the possible tumor region of WT. For better coverage, we expand the possible tumor regions by a few voxels with dilation. In the second phase, we construct a new density function in  (12) (see the following) according to the predicted and outer expanded tumor regions with higher densities for a new OMT, yielding enlarged tumor regions in the target cube while retaining unchanged nontumor regions. We then train Net1, Net2 and Net3 for WT, TC and ET, respectively, by the ResUnet algorithm. Consequently, the new OMT provided in Phase I is implemented analogously to a magnifying glass that enhances viewing and marking the brain tumor segmentations in Phase II.

### Phase I

**Figure 2:**
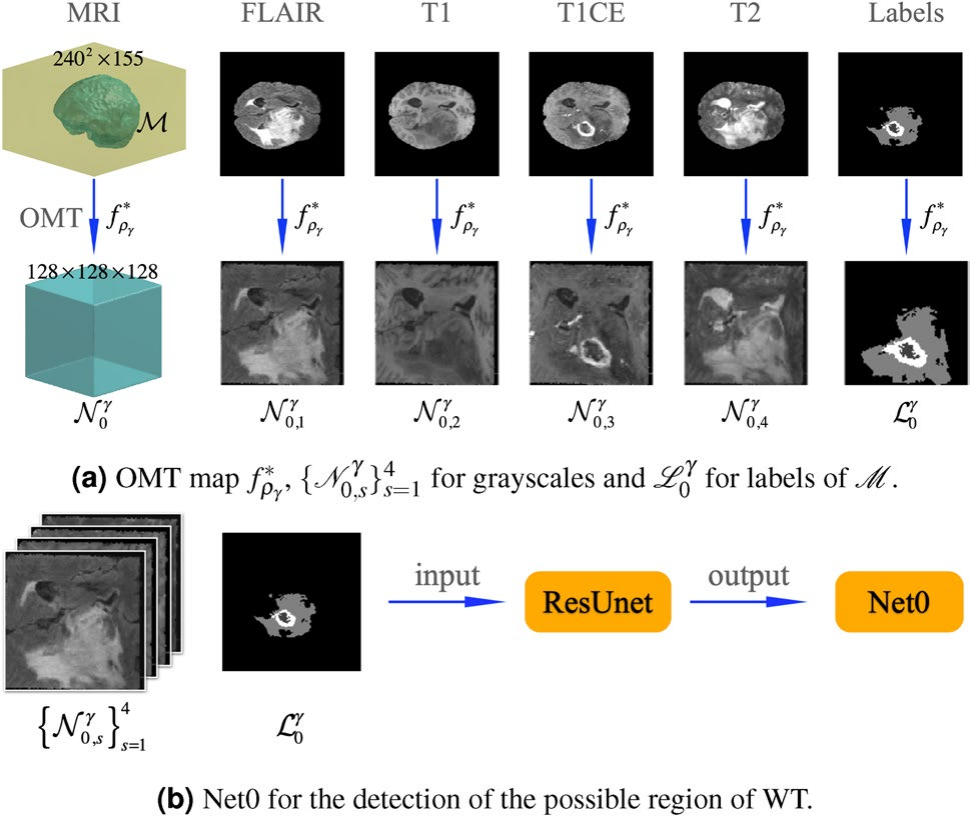
The procedure of Phase I. (**a**) Compute the OMT map $f_{\rho _\gamma }^*$ from $\mathcal {M}$ to $\mathcal {N}_0^\gamma$ and construct $\{\mathcal {N}_{0,s}^\gamma \}_{s=1}^4$ of $128 \times 128 \times 128 \times 4$ grayscale values for FLAIR, T1, T1CE, and T2 and $\mathcal {L}_0^\gamma$ with labels of $128\times 128\times 128$ for $\mathcal {M}$ by MRI. (**b**) Input format $\{\mathcal {N}_{0,s}^\gamma \}_{s=1}^4$ and $\mathcal {L}_0^\gamma$ for ResUnet and the trained Net0.

We first construct training tensors by using the OMT algorithm with the density $\rho _{\gamma }(v)$, as in (11). We compute the OMT map $f_{\rho _\gamma }^*$ with Algorithm 2 from $\mathcal {M}$ to a $128\times 128\times 128$ cube $\mathcal {N}_0^\gamma$. Then, we construct four $128\times 128\times 128$ tensors $\{\mathcal {N}_{0,s}^\gamma \}_{s=1}^4$, one $128\times 128\times 128$ tensor $\mathcal {L}_0^\gamma$ corresponding to the grayscales of $\mathcal {M}\subseteq I_s$, $s=1,\ldots ,4$, and labels in $\mathcal {M}\subset \mathcal {L}$, as shown in Fig. 2a. The constructed procedure is as follows: via the OMT map $f_{\rho _\gamma }^*$, we define the grayscale and the label on each voxel $\mathtt{u}\in \mathcal {N}_{0,s}^\gamma$ and $\mathcal {L}_0^\gamma$ by $f_{\rho _\gamma }^{*-1}(c_\mathtt{u})$ in some voxels $\mathtt {v}$ of $I_s$ and $\mathcal {L}$, respectively, where $c_\mathtt {u}$ is the center of $\mathtt {u}$, for $s=1,\ldots ,4$. Then, as shown in Fig. 2b, we use ResUnet to train Net0 with $\{\mathcal {N}_{0,s}^\gamma \}_{s=1}^4$ and $\mathcal {L}_0^\gamma$ as the input tensors, where $\mathcal {L}_0^\gamma$ labels are $\mathbf {0}$ and $\mathbf {1}$ for normal and WT regions, respectively, in which $\mathbf {0}=\{0\}$ and $\mathbf {1} = \{2,1,4\}$ form the $240^2\times 155$ MRI image.

### Phase II

For a given training brain image computed by Phase I, we expand the possible tumor regions of WT by *m* voxels with morphology dilation, denoted by $\mathbb {T}\subseteq \mathcal {M}$. Let $\rho _\gamma (v)$ be a step-like function defined as

12 $$\begin{aligned} \rho _\gamma (v) = {\left\{ \begin{array}{ll} \exp (\gamma \overline{I}_1(i,j,k)), &{} \text {if }v\in \overline{I}_1(i,j,k)\subseteq \mathbb {T}, \\ 1.0, &{} \text {if }v\notin \mathbb {T}. \end{array}\right. } \end{aligned}$$

Then, we construct a new smooth density function using the image filtering technique by convolving $\rho _\gamma (v)$ in (12) with a $m\times m\times m$ blur box tensor, as follows:

13 $${\tilde {\rho _{\gamma}}} (v) \leftarrow {\rho_{\gamma}} (v) \otimes \frac {{\mathbbm{1}}_{m\times m\times m}}{m^3}, \quad v \in {\mathcal {M}}.$$

**Figure 3:**
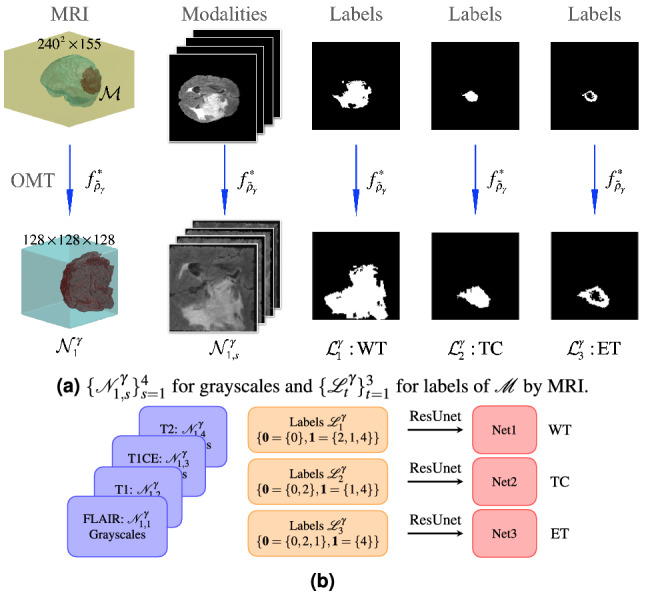
The procedure of Phase II. (**a**) Compute OMT $f_{\widetilde{\rho }_\gamma }^*$ from $\mathcal {M}$ to $\mathcal {N}_1^\gamma$ and $\{\mathcal {N}_{1,s}^\gamma \}_{s=1}^4$ of $128 \times 128 \times 128 \times 4$ grayscale values for $\{I_s\}_{s=1}^4$. Let $\mathcal {L}_1^\gamma$, $\mathcal {L}_2^\gamma$ and $\mathcal {L}_3^\gamma$ be labels of $128 \times 128 \times 128 \times 3$ for $\mathcal {M}$ corresponding to WT, TC and ET, respectively, where $\widetilde{\rho }_\gamma$ is given in (13). (**b**) Input format $\{\mathcal {N}_{1,s}^\gamma \}_{s=1}^4$ with $\mathcal {L}_1^\gamma$, $\mathcal {L}_2^\gamma$ and $\mathcal {L}_3^\gamma$ for ResNet and the trained Net1, Net2 and Net3 models for WT, TC and ET, respectively.

As shown in Fig. 3a, we compute the OMT map $f_{\widetilde{\rho }_\gamma }^*$ from $\mathcal {M}$ to a $128\times 128\times 128$ cube $\mathcal {N}_1^\gamma$ and construct four $128\times 128\times 128$ tensors $\{\mathcal {N}_{1,s}^\gamma \}_{s=1}^4$ corresponding to the grayscale values of $\mathcal {M}\subseteq I_s$ via OMT $f_{\widetilde{\rho }_\gamma }^*$, as in Phase I. Furthermore, we construct three $128\times 128\times 128$ tensors $\mathcal {L}_1^\gamma$, $\mathcal {L}_2^\gamma$ and $\mathcal {L}_3^\gamma$ associated with the labels of $\{\mathbf {0} = \{0\}, \mathbf {1}=\{2,1,4\}\}$, $\{\mathbf {0} = \{0,2\}, \mathbf {1}=\{1,4\}\}$, and $\{\mathbf {0} = \{0,2,1\}, \mathbf {1}=\{4\}\}$, respectively, of $\mathcal {M}$ by MRI. Here, $\mathcal {L}_1^\gamma$, $\mathcal {L}_2^\gamma$ and $\mathcal {L}_3^\gamma$ with $\mathbf {0}$ and $\mathbf {1}$ elements are the new labels of WT, TC, and ET, respectively. Then, as shown in Fig. 3b, we use ResUnet to train Net1, Net2 and Net3 for WT, TC and ET, respectively, where $\{\mathcal {N}_{1,s}^\gamma \}_{s=1}^4$ applies $\mathcal {L}_1^\gamma$, $\mathcal {L}_2^\gamma$ and $\mathcal {L}_3^\gamma$ as the initial input tensors.

### Net0 and Net1–Net3 for validation

**Figure 4:**
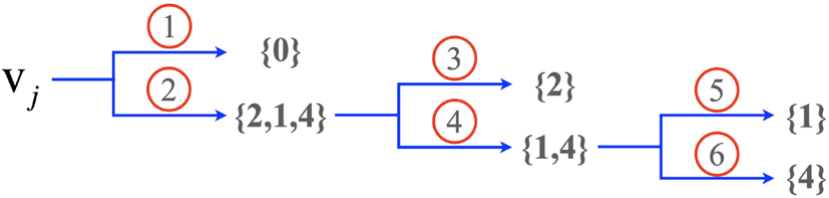
Flowchart of predicting the label of $\mathtt {v}_j\in \mathcal {M}\subset \mathcal {L}_*$.

Once we have computed Net0 and Net1–Net3 in Phase I and Phase II, respectively, we use Net0 to detect the possible tumor region of $\text {WT}=\{2,1,4\}$ with the density function $\rho _{\gamma }(v)$ defined in (11) and cover WT by *m* voxels with dilation, that is, $\mathbb {T}\subseteq \mathcal {M}$, and construct a new density function $\widetilde{\rho }_\gamma$, as in (12) and (13). We compute four $128\times 128\times 128$ cubes $\{\mathcal {N}_{1,s}^{\gamma }\}_{s=1}^4$ for the grayscale values of FLAIR, T1, T1CE and T2 via OMT $f_{\widetilde{\rho }_{\gamma }}^*$ and use Net1, Net2 and Net3 to validate three $128\times 128\times 128$ cubes, $\mathcal {L}_1^{\gamma }$, $\mathcal {L}_2^{\gamma }$ and $\mathcal {L}_3^{\gamma }$, for the predicted labels. Here, each voxel $\mathtt {u}_i^t$ in $\mathcal {L}_t^{\gamma }$, $t=1,2,3$, is predicted by a probability $0\leqslant \tilde{p}_i^t \leqslant 1$. The validation of a brain image in the validation set proceeds as follows. Let $\mathcal {L}_*\supset \mathcal {M}$ be a $240\times 240\times 155$ cuboid with each voxel in $\mathcal {L}_*$ set to $\{0\}$.

i. For each voxel $\mathtt {v}_j\in \mathcal {M}\subset \mathcal {L}_*$, let $f_{\widetilde{\rho }_{\gamma }}^{*-1}(c_{\mathtt {u}_i^t})\in \mathtt {v}_j$ for $t=1,2,3$, where $c_{\mathtt {u}_i^t}$ is the center of $\mathtt {u}_i^t$ for $i=1, \ldots , n(j) \ne 0$, and let $f_{\widetilde{\rho }_{\gamma }}^{*-1}(c_{\mathtt {u}_k^t})$ be the closest to the center of $\mathtt {v}_j$ if $n(j) = 0$. Then, we define the probability $p_j^t$ of $\mathtt {v}_j$ as
  14 $$\begin{aligned} p_j^t = {\left\{ \begin{array}{ll} \sum _{i=1}^{n(j)} \tilde{p}_i^t/n(j), &{} \text {if }n(j) \ne 0, \\ \tilde{p}_k^t, &{} \text {if }n(j) = 0. \end{array}\right. } \end{aligned}$$
ii. For $t=1,2,3$, at each voxel $\mathtt {v}_j\in \mathcal {M}$:
  Net1 evaluates $\mathbf {0} = \{0\}$ if $\textcircled {\small {1}}$ $p_j^1 < 1/2$ and $\mathbf {1}=\{2,1,4\}$, if $\textcircled {\small {2}}$ $p_j^1 \ge 1/2$;
  Net2 evaluates $\mathbf {0} = \{0,2\}$ if $\textcircled {\small {3}}$ $p_j^2 < 1/2$ and $\mathbf {1}=\{1,4\}$, if $\textcircled {\small {4}}$ $p_j^2 \ge 1/2$;
  Net3 evaluates $\mathbf {0} = \{0,2,1\}$ if $\textcircled {\small {5}}$ $p_j^3 < 1/2$ and $\mathbf {1}=\{4\}$ and if $\textcircled {\small {6}}$ $p_j^3 \ge 1/2$.
iii. The voxel $\mathtt {v}_j\in \mathcal {M}\subset \mathcal {L}_*$ is labeled by the procedure in Fig. 4.

In (ii), we see that for each voxel $\mathrm {v}_j$ in $\mathcal {M}$, we utilize the multiple values $p_i^t$ and $i=1,\ldots ,n(j)$ on the cube to define the most likely probability, which can be used to make a more precise evaluation of the label prediction.

**Figure 5:**
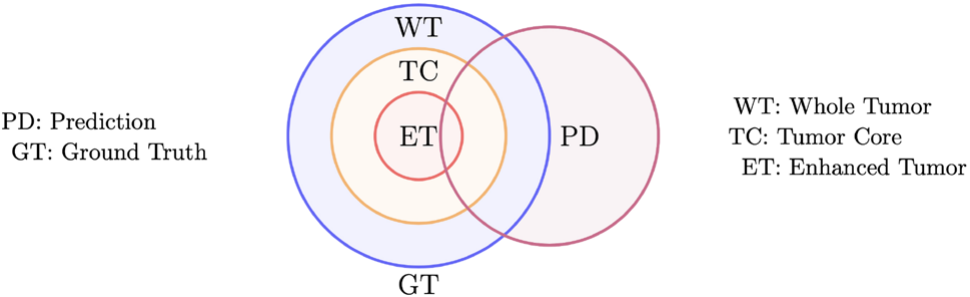
The relationships between WT, TC and ET of the ground truth and prediction.

Now, we denote $\mathrm {GT}$ as the ground truth of $\mathrm {WT}\supset \mathrm {TC}\supset \mathrm {ET}$ and $\mathrm {PD}$ as the prediction of $\mathrm {WT}$, $\mathrm {TC}$ and $\mathrm {ET}$, by (i)–(iii) above. The associated relationship between sets of GT and PD is plotted in Fig. 5. Let $\mathrm {GT}^c$ and $\mathrm {PD}^c$ be the complementary sets of $\mathrm {GT}$ and $\mathrm {PD}$, respectively. We consider the confusion matrix as

**Table:** 

	$\mathrm {GT}$	$\mathrm {GT}^c$
$\mathrm {PD}$	$\mathrm {TP}$ (true positive)	$\mathrm {FP}$ (false positive)
$\mathrm {PD}^c$	$\mathrm {FN}$ (false negative)	$\mathrm {TN}$ (true negative)

Here, we recall the following metrics^1^ for numerical experiments:

15 $$\begin{aligned} \mathrm {Dice} = \frac{2|\mathrm {GT} \cap \mathrm {PD}|}{|\mathrm {GT}|+|\mathrm {PD}|},\ \mathrm {Sensitivity} = \frac{|\mathrm {TP}|}{|\mathrm {TP}|+|\mathrm {FN}|},\ \mathrm {Specificity} = \frac{|\mathrm {TN}|}{|\mathrm {TN}|+|\mathrm {FP}|},\ \mathrm {Precision} = \frac{|\mathrm {TP}|}{|\mathrm {TP}|+|\mathrm {FP}|}. \end{aligned}$$

Furthermore, if we define $\text {GT}_p$ and $\text {PD}_p$ as the probability density tensors of GT and PD, respectively, we can define

16 $$\text {Loss function} = \text {Dice loss} + \text {Cross entropy loss} \equiv \left( 1-\frac{2\sum _i (\text {GT}_p \cdot \text {PD}_p)_i}{\sum _i (\text {GT}_p +\text {PD}_p)_i} \right) - \sum _{i} (\text {GT}_p)_i \log (\text {PD}_p)_i.$$

The Dice loss in (16) can help to check the convergence of the training procedure for WT, TC and ET vs. epochs by the ResUnet algorithm.

### Improvement in dice scores with mesh refinement and ensemble voting postprocessing

In this subsection, we propose two methods to improve the Dice scores of WT, TC, and ET. One is mesh refinement on the WT region for the OMT map, and the other is ensemble voting postprocessing.

- *Mesh refinement* With the merits of 2P-ResUnet-OMT, the density distribution of possible tumor regions in a brain image computed by Phase I can be enlarged with finer meshes and can be better viewed in Phase II for ResUnet training. One of the most important features of the OMT map is that the density can be increased in the region of interest, and then the region can be remeshed by the mesh refinement technique. In this way, due to the mass-preserving property of OMT, the region of interest can be enlarged in the cube, which enables ResUnet to learn more efficiently and achieve high-performance prediction results.
- *Ensemble voting postprocess* We propose an ensemble voting postprocessing approach to determine the final labels in the brain image for validation. The main purpose of this postprocessing step is to modify the probability $p_i^t$, $t=1,2,3$, in Steps (i)–(iii) of paragraph “Net0 and Net1–Net3 for Validation”. We first select the three best models $\{ \text {Net1}_\nu , \text {Net2}_\nu , \text {Net3}_\nu \}_{\nu = 1}^3$ for WT, TC and ET from the training procedure. For each $128\times 128\times 128$ brain tensor $(\mathcal {R}_0)$ for validation, we further build four $128\times 128\times 128$ tensors with 90 degree counterclockwise rotations $(\mathcal {R}_1)$, mirroring from the left to the right $(\mathcal {R}_2)$, mirroring from the top to the bottom $(\mathcal {R}_3)$ and mirroring from the left to the right followed by a 90 degree counterclockwise rotation $(\mathcal {R}_4)$.
  For $t=1,2,3$, at each voxel $\mathrm {u}_i^t$ in $\mathcal {R}_0, \ldots , \mathcal {R}_4$, $\text {Net}t_1$, $\text {Net}t_2$ and $\text {Net}t_3$ can predict 15 probabilities $\{p_{\mu ,\nu }^t(i) \mid \mu =0,\ldots , 4, \nu =1,2,3\}$. We compute
  17 $$\begin{aligned} \text {Dice}([p_{0,1}^t], [p_{\mu ,\nu }^t]) \equiv D_{\mu ,\nu },\quad \alpha _{\mu ,\nu }^t = {\left\{ \begin{array}{ll} 1, &{} D_{\mu ,\nu } \geqslant 0.8,\\ 0, &{} \text {otherwise} \end{array}\right. } \end{aligned}$$
  and
  18 $$\begin{aligned} p_i^t = \sum _{\nu =1}^3 \sum _{\mu =0}^4 \alpha _{\mu ,\nu } p_{\mu ,\nu }^t(i) / N, \end{aligned}$$
  where $[p_{\mu ,\nu }^t]=\text {The set of }\{0,1\}\text{ by taking Gaussian symbols of }p_{\mu ,\nu }^t(i)$ and $N=\sum _{\nu =1}^3 \sum _{\mu =0}^4 \alpha _{\mu ,\nu }$. Then, we perform the conversion in Steps (i)–(iii) to convert the associated labels back to the brain image.
  The various rotations $\mathcal {R}_1, \ldots , \mathcal {R}_4$ of the brain tensor $\mathcal {R}_0$ constructed above indeed help to improve the Dice scores with the ensemble voting technique developed in (17) and (18).

## Results and discussions

Based on the CNN technique, the U-Net algorithm is designed to learn an effective network from training data using an optimization process that requires decreasing the model error of the loss function on the training and validation sets. We adopt the ResUnet algorithm^22^ and set the hyperparameters as follows: encoder depth: 3, initial learning rate: $\alpha _0 = 1.0\times 10^{-4}$, learning rate drop factor: $F=0.95$, learning rate drop period: $P = 10$, $L_2$-regularization: $1.0\times 10^{-4}$, and minimum batch size: 8.

For the 1251 brain image samples in the BraTS 2021 challenge database^3,4,16^, we randomly fix 1000 samples for training and 251 for validation. Our utilized ResUnet is implemented in PyTorch and the Medical Open Network for AI (MONAI)^34^, and training is carried out on a server equipped with an NVIDIA Tesla V100S PCIe 32 GB$\times 4$ GPU.

### Partition number *p* in Algorithm 2

**Figure 6:**
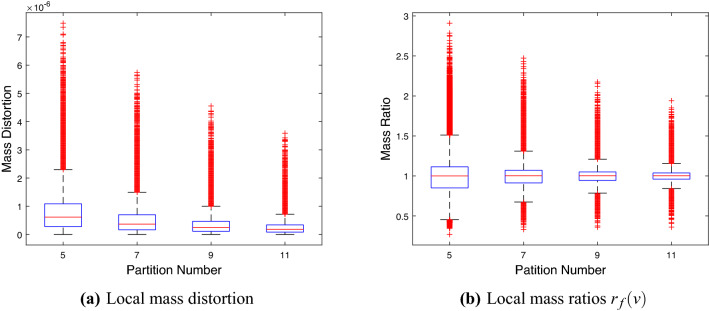
Statistical summary of the (**a**) local mass distortion and (**b**) $r_f(v)$ for all vertices of *BraTS0002* vs. the partition number *p* of homotopy.

**Figure 7:**
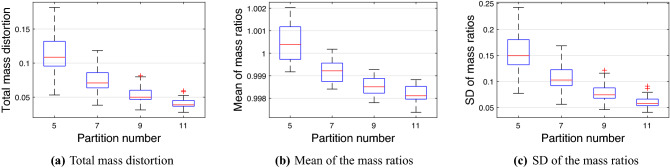
Statistical summary of the (**a**) $d_\mathcal {M}(f)$; (**b**) mean and (**c**) SD of $r_f(v)$ vs. the partition number *p* of homotopy for the first 1000 brain samples of BraTS 2021.

We select *BraTS0002* as an $\mathcal {M}$ from the BraTS 2021 dataset and compute the cubic V-OMT from $\mathcal {M}$ to $\mathcal {C}^3$ by Algorithm 2. In Fig. 6, we plot the statistical summary of the local mass distortion $\sum _{\tau \in \mathcal {N}(v)} |\rho (\tau )|\tau |-|f(\tau )||/4$, as in (10), and $r_f(v)$ for all $v\in \mathbb {V}(\mathcal {M})$ versus the partition number *p* of homotopy. In each box, the red centerline indicates the median, and the bottom and top edges of the box indicate the 25th and 75th percentiles, respectively. The dotted lines extend to the most extreme data points that are not considered outliers, and the outliers are represented separately with “+” signs. Furthermore, in Fig. 7, we also plot the statistical summary of the total mass distortion $d_\mathcal {M}(f)$ and the mean and standard deviation (SD) of $r_f(v)$ vs. the partition number *p* for the first 1000 brain samples from the BraTS 2021 dataset.

Figure 6 shows that when $p=11$, the cubic V-OMT between *BraTS0002* and $\mathcal {C}^3$ has the smallest local mass distortion and the closest local mass ratio to one. Moreover, in Fig. 7, when $p=11$, the first 1000 brain samples of BraTS 2021 have the smallest total mass distortion and the best mean and SD of the local mass ratios. Therefore, we choose $p=11$ in Algorithm 2.

### Dimension *m* of blur box tensor

We now discuss the dimension *m* of the blur box tensor in (13), which covers the WT region by *m* voxels. To choose a suitable number *m* for the covering voxels with dilation for WT, we apply the sensitivity and precision metrics defined in (15), in which $\mathrm {PD}$ denotes the prediction of {WT covered by *m* voxels with dilation} by Net0.

**Figure 8:**
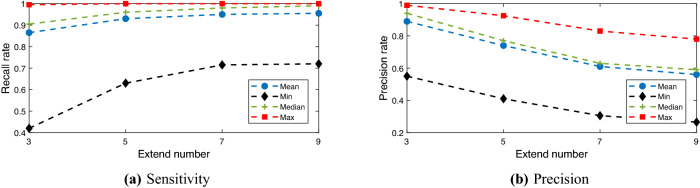
Mean, minimum, median and maximum values of the sensitivity and precision metrics of the WT validation vs. the numbers of covering voxels.

In fact, the sensitivity metric in (15) indicates how many voxels lie in the prediction, and the precision metric in (15) indicates how precise the prediction is. Thus, we want to make both the sensitivity and precision as large as possible. In Fig. 8, we plot the mean, minimum, median and maximum values of the sensitivity and precision metrics of the WT validation vs. the numbers of covering voxels with dilation. We find that $m=5$ is a suitable number to balance the sensitivity and precision values for the validation data.

For a fixed $m=5$, in Table 1, we list the mean, minimum, median, maximum and SD values of the transport costs, folding numbers and enlarged ratios for both the 1000 training samples and the 251 validation samples. The enlarged ratio is defined by $\text {(the ratio of WT in the cube)}/\text {(the ratio of WT in the raw data)}$.

**Table 1:** Mean and SD values of the transport costs, folding numbers and enlarged ratios of $f_{\widetilde{\rho }_1}^*$ from $\mathcal {M}$ to $\mathcal {C}^3$.

BraTS 2021	Transport cost		#Fold.		Enlarged ratio	
	Mean	SD	Mean	SD	Mean	SD
Validation (1000)	0.028	0.004	17.13	9.49	1.480	0.078
Testing (251)	0.029	0.003	18.62	9.24	1.444	0.079

In Table 1, we observe that the numerical results of the transport costs, folding numbers and enlarged ratios for the 1000 training and 251 validation samples computed by $f_{\widetilde{\rho }_1}^*$ in (13) are in line with what we expected.

### Dice scores and loss functions

**Figure 9:**
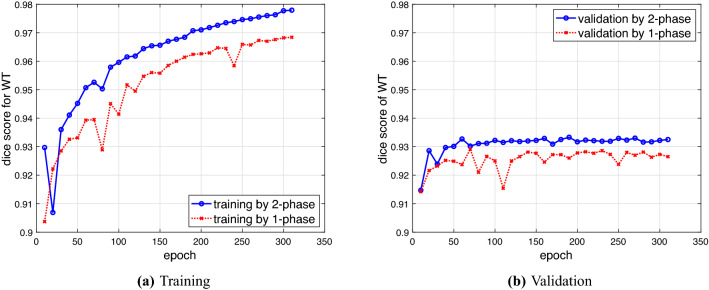
Dice scores of WT for training and validation by 2P- and 1P-ResUnet-OMT.

**Figure 10:**
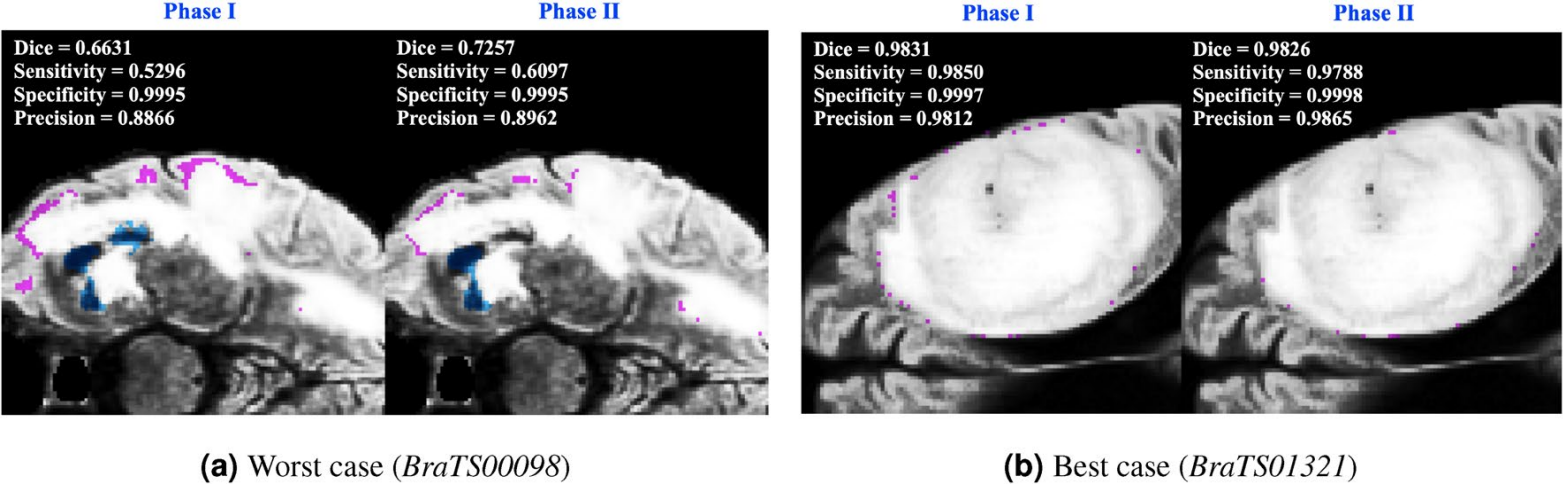
FP (purple area) and FN (blue area) for WT segmentation predicted by Phases I and II for (**a**) the worst case (*BraTS00098*) and (**b**) the best case (*BraTS01321*).

We first compare 2P-ResUnet-OMT developed in Section “Two-Phase ResUnet with OMT for Training and Validation” with one-phase ResUnet-OMT (1P-ResUnet-OMT), i.e., the density functions of (11) with $\gamma =1.0$ and 1.5 are used for training Net1. We learn Net1 by using 2P- and 1P-ResUnet-OMT with 300 epochs. In Fig. 9a,b, we plot the Dice scores of WT for training and validation by 2P- and 1P-ResUnet-OMT, respectively. We observe that for both the training and validation scores, 2P-ResUnet-OMT is obviously much better than 1P-ResUnet-OMT. Therefore, in the following numerical experiments, we prefer to adopt 2P-ResUnet-OMT. Furthermore, in Fig. 10, we compare the prediction results of FP (purple area) and FN (blue area) using Phases I and II, respectively, for the worst (*BraTS00098*) and best (*BraTS01321*) Dice performance of real brain images. For the worst case, we see from Fig. 10a that Phase II significantly reduces the ratios of FP and FN and has a considerable improvement in Dice, sensitivity, and precision. For the best case shown in Fig. 10b, Phases I and II have only a slight difference between the four metrics in (15). Overall, Phase II actually reinforces the examples of underperforming prediction accuracy by Phase I.

**Figure 11:**
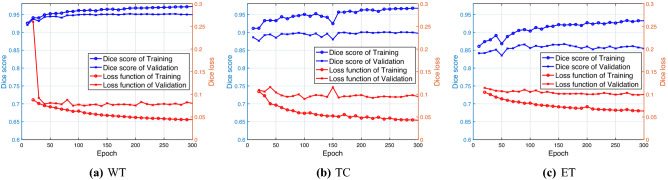
Dice scores (blue) and loss functions (red) for the training ($\circ$) and validation ($\times$) sets of WT, TC and ET vs. the epoch number.

To expand the training data in Phase II, we use three different density functions $\widetilde{\rho }_1(v)$, $\widetilde{\rho }_{1.5}(v)$ and $\widetilde{\rho }_{2}(v)$ for $v\in \overline{I}_1(i,j,k)\subseteq \mathcal {T}$, as in (13), to create 3000 augmented brain images for training. We now use 2P-ResUnet-OMT to train Net0 and Net1-Net3 on 3000 training samples. Then, we utilize them to obtain predictions on the 251 validation samples. In Fig. 11, we plot the Dice scores with blue “o” and “x” symbols and the loss functions with red “o” and “x” symbols vs. the epoch numbers for the training and validation sets of WT, TC and ET, respectively. Note that the Dice scores for WT, TC and ET are defined by (15), and the loss function is defined by (16). The predicted labels of WT, TC and ET in a brain image are evaluated by Steps (i)–(iii), which are precisely determined by the probability value $p_j^t=\sum _{i=1}^{n(j)} p_i^t / n(j)$, ($t=1,2,3$) in each voxel $\mathrm {v}_j\in \mathcal {M}$.

We see that the training and validation Dice scores for WT, TC and ET increase very quickly during the first 50 epochs but then do not increase significantly and reach (0.9720, 0.9673, 0.9330) and (0.9325, 0.8965, and 0.8614), respectively, after 300 epochs. On the other hand, the training and validation loss functions for WT, TC and ET decrease very quickly during the first 50 epochs and approach ($7.008 \times 10^{-2}$, $7.067 \times 10^{-2}$, and $8.678 \times 10^{-2}$) and ($8.006 \times 10^{-2}$, $9.487 \times 10^{-2}$, and $9.957 \times 10^{-2}$), respectively, after 300 epochs. The trends of both the Dice score and loss function value indicate the typical training and validation history. Thus, based on the clear tendency of the curves of the Dice scores and loss functions, in our experiment, we run ResUnet for 300 epochs.

### Merits of mesh refinement on the WT region

Based on the discussion of the merits of mesh refinement on the expanded WT region in the brain image, we compute OMT maps with the smooth density functions $\widetilde{\rho }_\gamma (v)$, $\gamma =1.0$, 1.5, 1.75, and 2.0 in (13) for 1000 brain samples to obtain 4000 augmented brain cubes and use ResUNet to train Net1–Net3. Furthermore, for validation, we compute 2P-OMT for 251 brain samples with the density function $\widetilde{\rho }_{1.75}(v)$ on the expanded WT region by Phase I with mesh refinement.

We train ResUnet for 300 epochs on 4000 augmented brain tensors. From epochs 10 to 300, for every 10 epoch, we validate the Dice scores on the 251 samples of validation data for WT, TC and ET. In Table 2, we show the top three validation Dice scores for WT at epochs 150, 170, and 130; for TC at epochs 140, 120, and 170; and for ET at epochs 100, 70, and 80 by Steps (i)–(iii) in the previous section. The corresponding training Dice scores for WT, TC and ET are listed in the first three columns of Table 2. We see that the validation Dice scores for WT, TC, and ET for the brain image reach 0.93469, 0.90251 and 0.86912, respectively, which is a satisfactory result.

**Table 2:** Dice scores of WT, TC and ET for the brain image with mesh refinement on the training and validation sets.

$$\left. \begin{array}{c} \text{ Epochs } \\ \text{(WT, } \text{ TC, } \text{ ET) } \end{array} \right.$$	Dice scores					
	Training			Validation		
	WT	TC	ET	WT	TC	ET
(150, 140, 100)	0.95388	0.95411	0.90912	0.93451	0.90251	0.86912
(170, 120, 70)	0.95363	0.95038	0.90496	0.93469	0.90167	0.86720
(130, 170, 80)	0.95241	0.95475	0.89994	0.93355	0.90187	0.86697

### Dice scores with ensemble voting postprocessing

In this subsection, we show the improvement in Dice scores with mesh refinement and the ensemble voting postprocessing approach to determine the final labels in the brain image for validation. We first select the three best models for WT, TC and ET at epochs (150, 170, 130), (140, 120, 170) and (100, 70, 80), respectively, from the training procedure, as shown in Table 2, and call them $\text {Net1}_\nu$, $\text {Net2}_\nu$, and $\text {Net3}_\nu$ for $\nu =1,2,3$.

**Figure 12:**
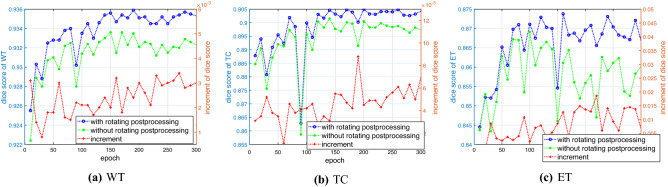
Dice scores with (blue line) and without (green line) the ensemble technique and increments (red line) for (**a**) WT; (**b**) TC; and (**c**) ET vs. the epoch number.

In Fig. 12a–c, we plot the histograms of the Dice scores with and without ensemble voting postprocessing in blue and green lines for WT, TC, and ET, respectively, vs. the epoch number. Furthermore, the associated increments of the Dice scores are plotted with red lines in Fig. 12a–c. We see that the Dice scores for WT, TC, and ET with the ensemble voting technique are much better than those without voting postprocessing. In addition, the Dice score curves for WT, TC and ET have a relatively stable upward trend.

In Table 3, we show the Dice, sensitivity, specificity, and 95th percentile of the Hausdorff distance^35^ (HD95) scores of 251 validation samples for WT, TC, and ET in brain images by $\text {Net1}_\nu$–$\text {Net3}_\nu$, $\nu =1,2,3$, with the ensemble voting technique. We see that $\text {Net1}_\nu$–$\text {Net3}_\nu$ with mesh refinement and ensemble voting postprocessing, as well as with the precise conversion of Steps (i)–(iii), significantly boosts the validation Dice scores (251) on BraTS 2021. This result is very promising for brain tumor detection and segmentation.

**Table 3:** Dice, sensitivity, specificity and HD95 scores on 251 validation samples for WT, TC and ET with the mesh refinement and the ensemble voting techniques.

2P-ResUnet-OMT + mesh refinement + ensemble voting				
Validation	Dice score	Sensitivity	Specificity	HD95
WT	0.93705	0.93641	0.99947	6.0129
TC	0.90617	0.90886	0.99974	7.9799
ET	0.87470	0.86945	0.99984	9.8300

Based on the description on the BraTS homepage^12^, we believe that the 2021 dataset is rich enough and contains almost all valid brain image data from past datasets (BraTS 2017–2020). To compare our results with related works in the survey paper^12^, we list some comparable results and associated techniques of^12^, which utilize the BraTS 2018 and 2019 datasets, as shown in Table 4. From this comparison, the 2P-ResUnet-OMT is quite satisfactory based on the Dice score performance.

**Figure 13:**
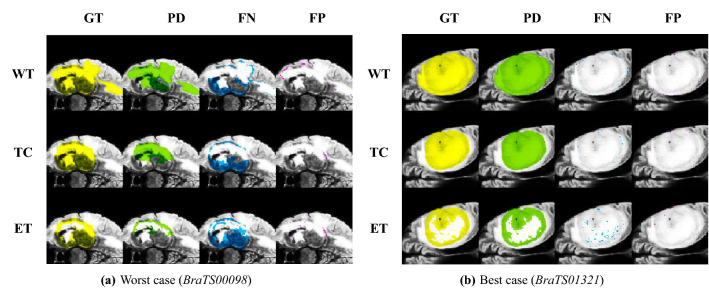
Segmentation of WT, TC and ET by Net1-Net3 of MRI images in FLAIR values for (**a**) the worst case (*BraTS00098*) and (**b**) the best case (*BraTS01321*).

Finally, to present visualization results of brain tumor segmentations, in Fig. 13, we show GT and PD predicted by $\text {Net1}_\nu$–$\text {Net3}_\nu$, $\nu =1,2,3$, and the corresponding FP and FN for (a) the worst case (*BraTS00098*) and (b) the best case (*BraTS01321*), respectively. In Fig. 13a, we observe that FN is mostly distributed in the area with low FLAIR values (dark gray area). This may be because we use the value of FLAIR as the density function to which OMT refers. Due to the mass-preserving property of OMT and the smaller density function value in the dark gray area, its proportion in the cube by the OMT is also smaller than that in the original image. Therefore, the predictions for this area are likely to be less accurate. The selection of a more effective density function to improve the prediction accuracy of this region is one of our main research topics in the near future.

**Table 4:** Comparison results of the preprocessing method, model architecture, and performance in some deep learning-based algorithms and BraTS datasets.

Paper	BraTS dataset	Preprocessing	Model architecture	Tumor type	Performance (DSC, SEN, SPE)
Ali et al.^36^	2019	Z-score normalization,cropping, rotation,and mirroring	Ensemble of a 3D CNN and a 3D U-net	WT TC ET	$(0.906,-,-)$ $(0.846,-,-)$ $(0.750,-,-)$
Sun et al.^37^	2018	Z-score normalization and cropping	3D FCN	WT TC ET	(0.900, 0.904, 0.995) (0.795, 0.751, 0.998) (0.771, 0.769, 0.998)
Sun et al.^37^	2019	Z-score normalization and cropping	3D FCN	WT TC ET	(0.890, 0.883, 0.995) (0.779, 0.762, 0.997) (0.761, 0.767, 0.998)
Akil et al.^38^	2018	intensity normalization (remove 1% highest and lowest intensities)	DCNN (Dense-MultiOCM)	WT TC ET	(0.862, 0.848, 0.995) (0.737, 0.710, 0.998) (0.710, 0.760, 0.998)
Aboelenein et al.^39^	2018	–	Hybrid two track U-net (HTTU-Net)	WT TC ET	(0.865, 0.883, 0.999) (0.808, 0.800, 0.998) (0.745, 0.780, 0.999)
Proposed	2021	Z-score normalization and two phase OMT	ResUnet with ensemble voting	WT TC ET	(0.937, 0.936, 0.999) (0.906, 0.909, 0.999) (0.875, 0.870, 0.999)

Here DSC, SEN, and SPE denote the dice score, sensitivity, and specificity, respectively.

## Conclusions

In this paper, we introduce 2P-ResUnet-OMT with density estimates for 3D brain tumor detection and segmentation. We first propose a cubic volume-measure-preserving OMT algorithm to compute an OMT map for transforming an irregular 3D brain image to a cube while preserving the local mass ratios and maintaining minimal deformation. Furthermore, OMT is bijective and minimizes the transport cost. The concept of expressing an irregular brain image as a cube with minimal distortion is proposed for the first time in this research field, and these cubes are typically adequate for the tensor input format of the ResUnet algorithm that creates validation networks. Representing 3D brain images as cubes significantly reduces the effective brain images from sizes of $240 \times 240 \times 155$ to cubes of sizes $128 \times 128 \times 128$ and preserves the global information of tumor features. This novel OMT preprocessing technique can save a large quantity of input data and reduce the computational time for training. In addition, the ensemble voting technique proposed in (17)–(18) and the robust conversion Steps (i)–(iii) of paragraph “Net0 and Net1–Net3 for Validation” from cubes ($128\times 128\times 128$) with predicted labels back to brain images ($240\times 240\times 155$) considerably increase the Dice scores for brain images compared to those for cubes on the 1,251 brain image samples.

One of the characteristics of the OMT map is that it can control the densities of tumor regions in brain images, and then, by mass-preserving OMT, the high-density areas can be enlarged in the cube so that the ResUnet algorithm can strengthen the cognition and learning in the high-density regions. In fact, 2P-ResUnet-OMT in the paragraph “Two-Phase ResUnet with OMT for Training and Validation” is designed for this purpose. Phase I first captures the possible region of WT and then covers this region with 5 voxels by dilation. Next, Phase II reconstructs new smooth density functions, as in (13), and performs mesh refinement on the range estimated by Phase I. With the advantage of the mass preservation of OMT, the portion of the possible WT region can be enlarged in the cube. Then, the ResUnet algorithm is utilized to train more effective Net1–Net3 models for tumor prediction and validation.

The Dice scores of WT, TC and ET by Net0 and $\text {Net1}_\nu$–$\text {Net3}_\nu$ for $\nu =1,2,3$, with mesh refinement and ensemble voting postprocessing reach 0.93705, 0.90617 and 0.87470 for validation, respectively. 2P-ResUnet-OMT with mesh refinement sufficiently utilizes the mass-preserving property to significantly improve the tumor detection and segmentation accuracy.

In future work, because an irregular 3D brain image needs to be represented by only a cube in our approach, we have much room to expand the augmented data with various density settings, such as in (12); these settings include rotating, mirroring, shearing and cropping and will allow for more opportunities to boost the prediction accuracy. In addition, we believe that for a 3D image provided by real 3D scanning instruments that may be developed in the future, the use of OMT to represent an irregular 3D object must retain the structure of the global information. This 3D OMT representation takes advantage of a precise conversion in the three directions in space and is beneficial to the input format of CNN algorithms. We believe this is a cross-trend research direction for medical images in the near future.
