# Author Correction: A novel 2-phase residual U-net algorithm combined with optimal mass transportation for 3D brain tumor detection and segmentation

**DOI:** 10.1038/s41598-022-12508-7

**Published:** 2022-06-07

**Authors:** Wen-Wei Lin, Jia-Wei Lin, Tsung-Ming Huang, Tiexiang Li, Mei-Heng Yueh, Shing-Tung Yau

**Affiliations:** 1 Nanjing Center for Applied Mathematics, Nanjing, 211135 People’s Republic of China; 2 Department of Applied Mathematics, National Yang Ming Chiao Tung University, Hsinchu, 300 Taiwan; 3 Department of Mathematics, National Taiwan Normal University, Taipei, 116 Taiwan; 4 School of Mathematics and Shing-Tung Yau Center, Southeast University, Nanjing, 210096 People’s Republic of China; 5 Department of Mathematics, Harvard University, Cambridge, USA

Correction to: *Scientific Reports* https://doi.org/10.1038/s41598-022-10285-x, published online 19 April 2022

In the original version of this Article, Wen-Wei Lin was incorrectly affiliated with ‘Nanjing Center for Applied Mathematics, Nanjing 211135, People’s Republic of China’. The correct affiliation is listed below.

Department of Applied Mathematics, National Yang Ming Chiao Tung University, Hsinchu 300, Taiwan.

The original Article has been corrected.

